# Two Provincial Hospitals

**Published:** 1907-03-09

**Authors:** 


### HOSPITAL ADMINISTRATION.

### CONSTRUCTION AND ECONOMICS.

### TWO PROVINCIAL HOSPITALS.

#### THE QUEEN'S HOSPITAL, BIRMINGHAM.

The Queen's Hospital, Birmingham, after sixty-five years, has acquired an addition to its present site, the purchase money having been mainly provided by a legacy of £5,000 granted by the Birmingham Corporation out of the estate of the late Thomas Best. An inspection of the hospital proves that in recent years the Committee have shown considerable ingenuity by modernising the existing buildings, and making them as light and airy as possible. The old wards, after reconstruction, present a comfortable appearance, and, although somewhat narrow, they are adequate to the purpose and may be regarded as satisfactory, with the exception of those on the upper floor where the windows should be extended to within a few inches of the ceiling, and the Guy's pattern of window-frame substituted for the existing ones. It is proposed to erect a new pavilion, which will provide accommodation for sixty additional beds and certain administrative offices. It is estimated that £30,000, of which a considerable amount has been promised, will be required to complete the new buildings. In Birmingham they have an excellent understanding between the various hospitals which prevents any clashing of appeals. The Queen's Hospital, for instance, has delayed the issue of its appeal until the completion of the building fund of the Women's Hospital, so that the raising of the requisite funds should not be a difficult matter. We hold this view after making a careful inspection of the Queen's Hospital, feeling that it is well administered, that the patients are exceedingly well cared for, and that the Committee deserve the support and confidence of the public. We venture to suggest that a small committee with some knowledge of printing and modern ideas should be appointed to select new type for, and to re-arrange, the annual report so as to make it as attractive and interesting a publication as possible. The present form is rather obsolete, and it would be well to include a plan of the existing hospital, and of the new site, with a few illustrations showing the principal work done in the wards of the institution. The report should also contain a building account, as, from the 1905 report, it is not possible to ascertain either the estimated expenditure on the new buildings, the amount of money promised, or the amount which has still to be raised. By giving life to the annual report public interest may be quickened and additional funds should flow in.

#### LEICESTER INFIRMARY.

The hundred and thirty-fourth report of the Leicester Infirmary is an interesting publication, full of information given in a form which makes it easy to understand the working, to review the progress, and to check the cost of the administration. Another year we would suggest that a few illustrations should be introduced, and that a plan of the whole institution as it exists to-day should be given. An interesting feature is the fact that the working classes contributed £7,479, a sum which represents nearly one-half the total expenditure (£15,912) in 1906. In addition to this magnificent contribution the working classes gave upwards of £3,200 to the convalescent home. Having regard to the wealth and constantly increasing prosperity of the town of Leicester, it is a little remarkable that the annual subscriptions should amount to only £3,568. In Leicester, as in smaller towns, as we pointed out last week, an active propaganda should be promoted amongst the middle class residents in favour of the hospital. Every man and woman ought to recognise the duty of rendering a little personal service in the days of health in the cause of the sick. This personal service can best be rendered by undertaking to raise one or two pounds a year at least in contributions for the local hospital. If Leicester were to start an Infirmary League, and to appoint captains of streets, each of whom would undertake to visit each house in the course of the year, and to receive on behalf of the Infirmary any small sums that the occupiers might be willing to give, there is no reason why the sum derived from annual subscriptions should not exceed the working class contributions by the end of the present year. The quality of the work done must be high, as the average stay per patient has been 21.7 days in 1906. This infirmary for many years past has been noted for the vigour of its administration. During the year upwards of £45,000 was raised, and expended in reconstruction works, including a new system of drainage, the modernising of the S.E. and S.W. wings, the erection of a new and perfected out-patient department, of a new boiler-house, refuse destructor and disinfecting-chamber, and the re-fitting of the culinary and housekeeping departments of the hospital. In addition to this, the whole system for heating and ventilating the buildings has been reconstructed. The Board have now undertaken the rebuilding of the S.E. wing, which will increase the total accommodation from 200 to 270 beds. The requisite funds have been speedily raised, and the Board have undertaken to build a new nurses' home and to renovate and improve the laundry. The successful financing of all these works is largely due to the energy and influence of Sir Edward Wood, the

Chairman, whose name appeared in the list of birthday honours, and who was elected Mayor of Leicester for the fourth time in November last. It is always pleasant to be able to record the vigorous and successful efforts of the inhabitants of a provincial city. Leicester will soon have a complete, modern hospital, as it has long possessed one of the most efficient of modern nurse-training schools. Economy is not lost sight of at Leicester, and a careful eye appears to be kept upon the expenditure. The report contains a budget for the out-patient and laundry departments, and also for the nurses' home. This is a good idea which might be usefully adopted by other hospitals. The means of the patients are systematically inquired into, and little abuse is stated to exist. The average cost of each in-patient in 1906 was £4 19s. 7d., and of each out-patient 2s. 3d. The cost of the out-patients is understated, because it does not include the cost of maintenance of the surgery, where a good many of the casualty cases are treated. The report, as we have said, is worthy of careful study, and must afford information on many useful points to all those who will devote a little time to its perusal.

